# DeepGaze III: Modeling free-viewing human scanpaths with deep learning

**DOI:** 10.1167/jov.22.5.7

**Published:** 2022-04-26

**Authors:** Matthias Kümmerer, Matthias Bethge, Thomas S. A. Wallis

**Affiliations:** 1 University of Tübingen, Tübingen, Germany; 2 Technical University of Darmstadt, Institute of Psychology and Centre for Cognitive Science, Darmstadt, Germany

**Keywords:** eye movements, saccades, saliency, probabilistic modeling, deep learning

## Abstract

Humans typically move their eyes in “scanpaths” of fixations linked by saccades. Here we present DeepGaze III, a new model that predicts the spatial location of consecutive fixations in a free-viewing scanpath over static images. DeepGaze III is a deep learning–based model that combines image information with information about the previous fixation history to predict where a participant might fixate next. As a high-capacity and flexible model, DeepGaze III captures many relevant patterns in the human scanpath data, setting a new state of the art in the MIT300 dataset and thereby providing insight into how much information in scanpaths across observers exists in the first place. We use this insight to assess the importance of mechanisms implemented in simpler, interpretable models for fixation selection. Due to its architecture, DeepGaze III allows us to disentangle several factors that play an important role in fixation selection, such as the interplay of scene content and scanpath history. The modular nature of DeepGaze III allows us to conduct ablation studies, which show that scene content has a stronger effect on fixation selection than previous scanpath history in our main dataset. In addition, we can use the model to identify scenes for which the relative importance of these sources of information differs most. These data-driven insights would be difficult to accomplish with simpler models that do not have the computational capacity to capture such patterns, demonstrating an example of how deep learning advances can be used to contribute to scientific understanding.

## Introduction

Humans, primates, and some other animals do not perceive all of their field of view in the same resolution. Instead, in the fovea, corresponding to a small central area in the field of view, receptor density in the retina is very high and decays in the periphery toward the boundary of the field of view. In order to gather high-resolution information about our visual environment, we have to make eye movements, directing the fovea toward whatever seems most relevant or interesting at that moment. When viewing static scenes, eye movements typically consist of *fixations*, where the gaze fixates a certain image location with only very little movement, and *saccades*, segments of high gaze velocity where the gaze moves from one fixation point to the next.

The principles governing such *scanpaths* of fixations have already been the subject of substantial research. Yarbus (1967) noticed that scanpaths differ visually when giving observers different tasks. The seminal model of Itti et al. (1998) provided a computational implementation of the feature integration theory of Treisman and Gelade (1980). Originally intended to explain effects on search duration in visual search, it was soon also applied to predict fixation locations when free-viewing images (Peters et al., 2005) and kickstarted the field of *saliency models*, computational models that predict a *saliency map* highlighting image areas that are likely to attract fixations. Originally, saliency models stayed close to the original feature integration theory and mainly made use of low-level features, for example, by simple pop-out detection (Itti et al., 1998), natural image statistics (Zhang et al., 2008), and information theory (Bruce & Tsotsos, 2009). Over time, for many researchers, saliency became synonymous with free-viewing fixation prediction, and models started to include high-level information such as object locations (Judd et al., 2009); for an extensive overview of saliency models, see Borji and Itti (2013). With the advent of deep learning, transfer learning from deep features massively boosted prediction performance (Kümmerer et al., 2015a, 2017). Today, all high-performing saliency models make use of deep features transferred from other computer vision tasks.

However, while saliency models only predict the spatial fixation distribution when viewing still images, the fixations of a scanpath are known to be highly dependent on each other. Oculomotor biases influence saccade amplitudes and directions, but also task and memory can affect the order in which image regions are scanned and whether a certain image region is explored at all. *Scanpath models* try to take these effects into account. By predicting not only spatial fixation locations (e.g., by the means of a saliency map) but also whole scanpaths of fixations, they can model the effect of earlier fixations in a scanpath on later fixations and therefore the exploration behavior. While the field of scanpath modeling has not received as much attention as the field of saliency modeling, recent years have seen a substantial number of models of scanpath prediction, mostly focused on free-viewing scanpaths (see Kümmerer & Bethge, 2021, for an extensive overview of models of scanpath prediction). The model of Itti et al. (1998) modeled sequences of fixations via a winner-takes-all (WTA) module that got inhibited after each fixation to encourage a new fixation. Boccignone and Ferraro (2004) proposed to model scanpaths as a constrained Lévy flight, that is, a random walk where the step length follows a Cauchy–Lévy distribution and therefore is very heavy tailed. Engbert et al. (2015) and Schütt et al. (2017) proposed a mechanistic model of scanpaths that implemented an attention and an inhibition mechanism with certain decay times to predict a sequence of fixations. Le Meur and Coutrot (2016) combined a saliency map with saccade direction and amplitude biases. Adeli et al. (2017) took inspiration from neuroscience and transformed a retinotopic saliency map to superior colliculus space where the fixation selection was implemented. Clarke et al. (2017) proposed the saccadic flow baseline for capturing oculomotor biases independent of image content. Assens et al. (2017) used deep neural networks to predict different spatial fixation distributions depending on the amplitude of the previous scanpath history and combined this with a bias toward short saccades to generate a scanpath. Xia et al. (2019) built a variational autoencoder model of image statistics over the previous fixations and selected fixations where the internal model had the largest reconstruction error. Sun et al. (2019) used recurrent neural networks to model attention and suppression to certain spatial and semantic features over a sequence of fixations. Yang et al. (2020) used inverse reinforcement learning to train a policy in a deep learning model that mimics human scanpaths in visual search. Schwetlick et al. (2020) extended the attention and inhibition mechanism in the model of Engbert et al. (2015) to include perisaccadic attentional dynamics.

Recently, we conducted an extensive review and benchmark of free-viewing scanpath models and found that only few scanpath models reach higher performance than state-of-the-art spatial saliency models (Kümmerer & Bethge, 2021). This was even the case when the scanpath models were modified to use these state-of-the-art saliency models as their internal priority map—indicating that whatever conditional information these models attempt to capture made predictions worse than sampling independent locations from the saliency map. It appears that many of the proposed mechanisms of fixation selection, although often firmly based in results from neuroscience and vision science, do not account for the most important effects governing dependencies between fixations in a scanpath.

Moreover, it is difficult to even estimate how well sequences of fixations could be predicted in theory. In the case of the two-dimensional spatial fixation distribution p(x,y∣I) for an image I, it is fairly simple to estimate the ground truth distribution, for example, with Gaussian kernel density estimates from empirical data. In the case of scanpath prediction, however, the relevant distribution $p(x_{0},y_{0},\cdots ,x_{N},y_{N}|I)$ or $p(x_{N},y_{N}|x_{0},y_{0},\cdots ,x_{N-1},y_{N-1},I)$ is very high-dimensional. Estimating it from empirical data using a kernel density would require massive numbers of scanpaths to be recorded for each image in order to capture all possible dependencies between fixations.

Therefore, there is a need for high-performing computational models of scanpath prediction. Even if such a model were a complete black box, it would provide value for the field by estimating how well scanpaths can be predicted in theory. While learning, it can combine information from many different scanpaths on many different images and eventually provide a better estimate of the empirical scanpath distribution for each image than would be possible from the available ground truth data for this image alone. Interpretable models of scanpath prediction then can use such high-performing models to put their own prediction performance in perspective and quantify how relevant the proposed mechanisms are for fixation selection.

To that end, we present *DeepGaze III*, a deep learning–based model of free-viewing scanpath prediction. DeepGaze III is an extension of DeepGaze II, our state-of-the-art model of spatial fixation prediction. We fit the parameters of DeepGaze III on scanpaths of human subjects free-viewing natural scenes. We find that the model substantially outperforms the previous state-of-the-art scanpath models on free-viewing human scanpath data. DeepGaze III reproduces several key statistics of human scanpaths, such as the saccade amplitude and direction distribution. Using a series of ablation studies and other modifications of the model architecture, we gain insights into several factors that affect the fixation selection process. We analyze the effects of image content and scanpath history on the fixation selection process as well as the possible kinds of interactions between them; we disentangle the overall tendency to fixate close to the center of the image into the contributions from photographer bias, oculumotor bias, and remaining central fixation bias; and we quantify how the contribution of different factors on gaze placement changes over the course of a scanpath.

## Theory

One cannot expect to perfectly predict the scanpath of an observer on a given image. First, we don't expect human scanpaths themselves to be perfectly deterministic. The brain state of each observer will be different, due, for example, to differing memory and interests, and also, there is noise in neural firing and in the oculomotor system, which can create additional stochasticity in the scanpaths. Second, even if scanpaths were completely deterministic, the space of possible scanpaths is high-dimensional such that it is unlikely we will ever have enough data to perfectly learn the rules determining scanpaths.

For this reason, in this work, we approach the scanpath prediction problem using probabilistic generative modeling. Via the encoded probability distribution, probabilistic modeling gives our model the ability to express the diversity in possible scanpaths and their relative likelihood. Probabilistic modeling is a very well-established approach that has been applied before in the field of fixation prediction (Vincent et al., 2009; Barthelmé et al., 2013; Kümmerer et al., 2015b). Here, we are interested in modeling the distribution of scanpaths given an image and the initial central fixation $p(f_{1},f_{2},f_{3},\cdots ,f_{N}|f_{0},I)$. We condition on the seen image, because clearly, the content of the image will affect how observers move their gaze over the image. We also condition on the initial fixation, because the initial fixation in the data we model is not a free fixation made by the observer, but enforced through the experimental setup and will clearly affect the following fixations.

To make the full distribution over scanpaths $p(f_{1},f_{2},f_{3},\cdots ,f_{N}|f_{0},I)$ more tractable, we apply the chain rule. We split up the full distribution into a product of conditional probabilities, one for each fixation given all previous fixations in the scanpath:

$$\begin{array}{c} p\left(f_{1},f_{2},f_{3},\cdots ,f_{N}|f_{0},I\right)=\prod_{i=1}^{N}p\left(f_{i}|f_{0},\cdots ,f_{i-1},I\right) \end{array}$$

This approach has been applied in many contexts to make complex distributions more tractable, for example, in natural image statistics (Hosseini et al., 2010; Theis et al., 2012) and also for scanpath prediction (Schütt et al., 2017; Schwetlick et al., 2020; Malem-Shinitski et al., 2020). In the case of scanpath modeling, it is especially natural. Beyond being a mathematical trick to make the distribution more tractable, it resembles how we assume scanpaths are generated in the brain: Evidence from neuroscience (Kalesnykas & Sparks, 1996; Girard & Berthoz, 2005) suggests that while fixating a point in an image, the brain selects where to saccade to next by incorporating task, oculomotor biases, and memory. In other words, where we looked before influences where we might look next. This is captured in the conditional distributions for each fixation $p(f_{i}|f_{0},\cdots ,f_{i-1},I)$.

We can generate new scanpaths from the model by sampling from its distribution, making use of the chain rule decomposition. We start by sampling the first free fixation $f_{1}$ from $p(f_{1}|f_{0},I)$. Then we use the sampled fixation $f_{1}$ to sample the second free fixation $f_{2}$ from $p(f_{2}|f_{0},f_{1},I)$ and so on until we get a scanpath of the desired length. In fact, even without the mathematical justification, most models of scanpath prediction adhere to this principle by generating scanpaths fixation by fixation, where each previous fixation affects which future fixations might occur: Effectively, a scanpath forms a sequence of consecutive decisions.

In order to assess how well any given scanpath is predicted by the model, we can compute its log-likelihood $\log p(f_{1},f_{2},f_{3},\cdots ,f_{N}|f_{0},I)$. The log-likelihood is a principled measure of prediction performance (see Kümmerer et al., 2015b, for an extensive discussion of log-likelihood in the context of spatial fixation prediction). The product decomposition of the distribution transfers into a decomposition of the log-likelihood, which can be written as the sum over the conditional log-likelihoods for each fixation: $\log p\left(f_{1},f_{2},f_{3},\cdots ,f_{N}|f_{0},I\right)=\sum_{i=1}^{N}\log p\left(f_{i}|f_{0},\cdots ,f_{i-1},I\right)$. To make the log-likelihoods more comparable over scanpaths, it is useful to normalize by the length of the scanpath and therefore simply compute average log-likelihoods per fixation. This number quantifies how well each fixation is predicted on average. By scoring scanpath prediction as average log-likelihood per fixation, we effectively reduced scanpath prediction to the case of spatial fixation prediction. The only difference is that for each fixation, we do not evaluate the scanpath history independent distribution p(f∣I) but the conditional distribution $p(f_{i}|f_{0},\cdots ,f_{i-1},I)$.

See Kümmerer and Bethge (2021) for an extensive review of this way of scanpath modeling and how it relates to both nonprobabilistic scanpath models and spatial fixation prediction models.

## Model

**Figure 1.**
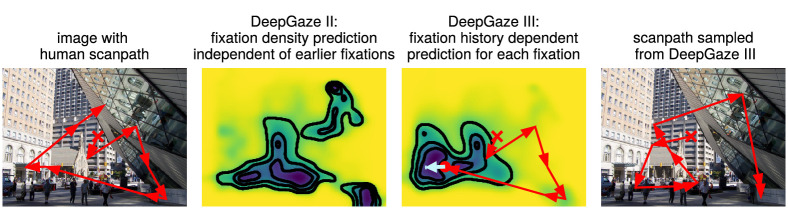
Humans explore still images in scanpaths consisting of fixations linked by saccadic eye movements (left). Static saliency models such as DeepGaze II predict the average fixation density over the image, independent of where a participant might have looked before (center left). DeepGaze III models human scanpaths by predicting a distribution over possible next fixations given a previous scanpath history and the image (center right). Iteratively sampling from this conditional distribution allows the sampling of new scanpaths from the model (right).

Our scanpath model DeepGaze III is a generative probabilistic model as discussed in the previous section. Given an image I and a partial scanpath $f_{0}=\left(x_{0},\;y_{0}\right),\;f_{1}=\left(x_{1},\;y_{1}\right),\;\cdots \;f_{i-1}=\left(x_{i-1},\;y_{i-1}\right)$ that a subject might have made so far, where x and y denote spatial fixation coordinates and the subscript indicates the fixation's number in the sequence, the model predicts a *conditional fixation distribution* $p(f_{i}|f_{0},\cdots ,f_{i-1},I)$ (Figure 1, center right). To make the model more tractable, we use only a limited number k of most recent fixations $p\left(f_{i}|f_{0},\cdots ,f_{i-1},I\right)=p\left(f_{i}|f_{i-k-1},\cdots ,f_{i-1},I\right)$ and assume that the conditional probability does not depend on how many fixations have been made before these most recent fixations, that is, $p\left(f_{i}|f_{i-k-1},\cdots ,f_{i-1},I\right)=p\left(f_{j}|f_{j-k-1},\cdots ,f_{j-1},I\right)$. For most results presented in this article, we use k=4, that is, DeepGaze III takes the four most recent fixations made by the subject into account—the current fixation and the three previous fixations.

**Figure 2.**
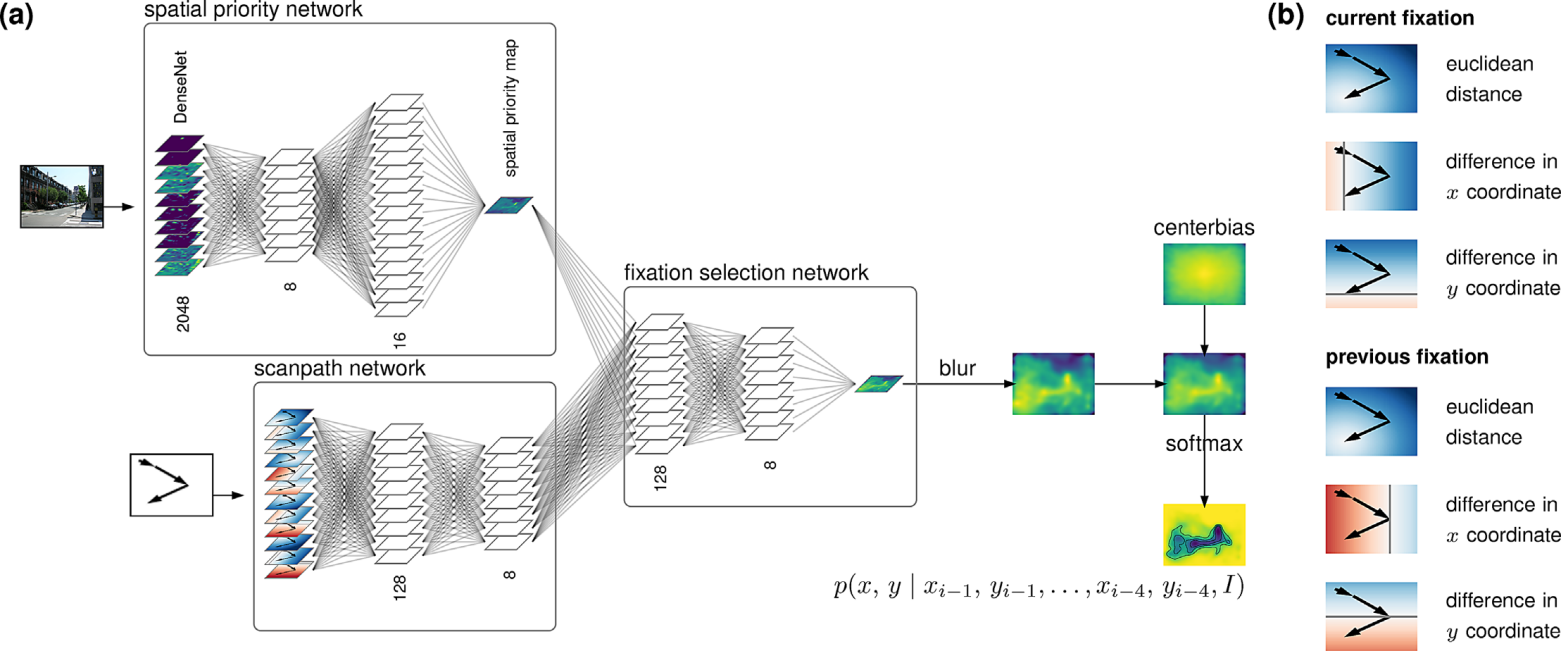
The DeepGaze III model. (a) Model architecture consisting of spatial priority network, scanpath network, and fixation selection network. A viewed image is processed with the spatial priority network to compute a spatial priority map. Information about the previous fixations a subject made is processed with the scanpath network and then combined with the spatial priority map in the fixation selection network. Finally, the prediction is blurred, combined with a center bias, and converted into a probability distribution by a softmax. (b) In order to make the model aware of the previous scanpath history, we encode the last four fixations of the scanpath history into three two-dimensional feature maps each. These feature maps are the Euclidean distance and difference in x and y coordinate to the encoded fixation. Here, we show the encoding feature maps for the last two fixations. In the feature map examples shown here, we superimpose the last three saccades with arrows. The current fixation location is in the bottom left and the previous fixation location is in the center right. Colors indicate the value of the feature map for each pixel, with blue indicating positive values and red indicating negative values. Gray lines indicate values of zero.

Figure 2 visualizes the DeepGaze III model architecture. The architecture of DeepGaze III can be seen as an extension of the architecture of the DeepGaze II model for spatial saliency prediction (Kümmerer et al., 2017). DeepGaze III receives as input an image (upper left) and the scanpath history (lower left) and outputs the conditional fixation distribution, which is a two-dimensional probability density and encodes where the model expects the subject to fixate next (lower right). The image is downscaled by a factor of 2 and processed with the convolutional part of the DenseNet 201 deep neural network (Huang et al., 2017). By extracting the activations for multiple layers from DenseNet for the given input image, we compute a deep representation of the image. More precisely, we use the layers denseblock4.denselayer32.norm1, denseblock4.denselayer32.conv1, and denseblock4.denselayer31.conv2, resulting in a total of 2,048 channels. These channels are concatenated and fed into the *spatial priority network*, a small readout network (Kümmerer et al., 2017) of three layers of 1×1 convolutions with 8 channels, 16 channels, and 1 channel, respectively. Before each layer, the input is normalized using LayerNorm (Ba et al., 2016), and after each layer, the softplus nonlinearity is applied. The spatial priority network outputs a single feature map that we call a *spatial priority map*, since it is supposed to encode the image-driven relevance of each image area. In many other models, it would be called a *saliency map*. We use the term *priority map* to emphasize that, while in this work we focus on free-viewing, the architecture itself is not restricted to that but could also incorporate task information.

Parallel to the spatial priority network is a *scanpath network* that processes the scanpath history. Each fixation that the model receives information from is encoded into three spatial feature maps encoding Euclidean distance and difference in x and y coordinates (Figure 2b). Our main model receives information about up to four previous fixations. The three feature maps for each of those four fixations are fed into four 1×1 convolutions that output 128 channels each. The outputs of the four convolutions are added up before being fed to the remaining part of the scanpath network. However, for early fixations in a scanpath, there might not yet be four previous fixations in the scanpath. In these cases, the convolutions for nonexisting fixations are simply ignored. The remaining part of the scanpath network is simply another 1×1 convolutional layer with layer norm and 16 output channels.

Finally, the output of spatial priority network and scanpath network is combined in the fixation selection network, again a network of 1×1 convolutions, this time with 128 channels, 8 channels, and 1 channel. The output of the fixation selection network is blurred with a Gaussian convolution and added to a center bias prediction after it is normalized with a softmax to output the conditional fixation distribution $p(x,\;y|x_{i-1},\;y_{i-1},\;\cdots ,\;x_{i-4},\;y_{i-4},\;I)$.

The learnable parameters of the model are the parameters of the three readout networks, the width of the Gaussian convolution, and the weight of the center bias. This results in a total number of 28,601 learnable parameters, most of which (20,488) are in the first layer of the spatial priority network. The channel sizes of the readout networks have been chosen via experimentation to allow sufficient computational capacity while keeping the number of parameters within a reasonable range. It would be possible to replace the three readout networks with just one readout network that receives a concatenation of image features and scanpath features. However, by splitting it into multiple modules, we can pretrain the spatial priority network without using scanpath data. This substantially reduces computation time, speeds up training, and also allows ablation studies.

## Methods

### Datasets

We use the publicly available MIT1003 dataset (Judd et al., 2009) to conduct our experiments. The MIT1003 dataset consists of 1,003 images of mainly color natural scenes with a longer side of 1,024 pixels. The authors of the dataset collected eye movements from 15 subjects with a 3-second presentation time and made scanpaths of fixations available. In training, we resized all images to be either 1,024 × 768 or 768 × 1,024 pixels in size to make batch processing easier. Unlike most works on static saliency, we do not exclude the initial forced central fixation from the scanpaths, since we want to allow models to model the influence of the first fixation on later fixations. However, the initial forced fixation is not included in evaluations; it is only used when informing models about previous fixations: We evaluate on exactly the same fixations as other works using MIT1003.

For pretraining, we also use the SALICON dataset (Jiang et al., 2015). It consists of mouse traces of human observers that explored images by means of moving a high-resolution “fovea” over an otherwise blurred image on a computer screen. SALICON includes mouse data for 10,000 training and 5,000 validation images. We use the 2015 version of the dataset.

To test model generalization to new images, we also evaluate prediction performance on the MIT300 dataset. It is the hold-out test set of the MIT/Tuebingen Saliency Benchmark (Judd et al., 2012; Kümmerer et al., 2018) and consists of eye movement data from 45 subjects on 300 images under conditions otherwise identical to the MIT1003 dataset. Unless stated otherwise, all results and visualizations presented below are from MIT1003.

We repeat some analyses on the CAT2000 dataset (Borji & Itti, 2015). It consists of 2,000 images with a resolution of 1,900 × 1,080 pixels evenly distributed over 20 categories such as indoor, outdoor, cartoons, art, and line drawings with eye movement data of 12 observers per image over a presentation time of 5 seconds. We apply the same preprocessing procedure as reported in Kümmerer and Bethge (2021), which mainly involves adding the missing initial central forced fixation to the scanpaths. As for MIT1003 and MIT300, we do not evaluate prediction performance on the initial forced fixation since it is not voluntary.

### Training

DeepGaze III is trained using maximum likelihood, that is, we maximize the average of the log-likelihood per fixation $\log p(f_{i}|f_{i-1},f_{i-2},f_{i-3},f_{i-4},I)$. More precisely, we average the log-likelihoods first over all fixations of an image and then over all images.

The training of DeepGaze III has multiple phases. First, the scanpath network is completely removed, converting the model into a purely spatial model that is essentially a version of DeepGaze II. In Phase 1, the spatial model is pretained on the SALICON dataset, using the spatial positions of the points in the mouse traces as proxy for fixations. In Phase 2, training of the spatial model is continued on the MIT1003 dataset, ignoring from each scanpath the initial central fixation, which is not a free fixation and therefore should not be predicted. In this phase and all subsequent phases, we use 10-fold cross-validation: We split the images into 10 parts, and for each cross-validation fold, we use eight parts for training, use one part for validation, and keep one testing part for our analyses. In subsequent phases of training on MIT1003, for each cross-validation fold, the model will be initialized from the corresponding fold of the previous phase, to ensure that the images of the test folds have never been seen by a model in earlier phases of training. Phases 1 and 2 allow us to efficiently pretrain the spatial priority network: In the spatial setting, all fixations for an image are evaluated on the same fixation density and therefore can be processed together. In Phase 3, for the first time, the scanpath network is included in the model. It is trained on the MIT1003 dataset, but the first layer of the spatial priority network (a fully connected layer from 2,048 inputs to 8 outputs, where most of the parameters of the model are located) is kept fixed. This allows us to find good values for the scanpath network and even allow the model to adapt the spatial priority network to some degree, while avoiding to run into overfitting problems in the spatial priority network. Finally, in Phase 4, the first layer of the spatial priority network is again included in training to fine-tune all trainable parameters. For training, the ADAM optimizer (Kingma & Ba, 2017) was used with a learning rate schedule specific for each training phase (see Table 1).

For evaluation and analyses on MIT1003, for each image, we use the parameters from the cross-validation fold, which contained this image in the testing split of the dataset. For evaluation on MIT300, we average predictions over all cross-validation folds.

For comparison purposes, we also train versions of DeepGaze III without the spatial priority network or without the scanpath network, resulting in image-independent and scanpath-independent baseline models.

To estimate the effect of random seeds and noise in training, the full training process was repeated eight times. All reported model scores are average scores over the repeated training runs.

Training on the CAT2000 dataset is identical to training on MIT1003. We make sure that categories are balanced over cross-validation folds. Due to the larger dataset, both in terms of image size and number of fixations, we did not repeat the training multiple times on the CAT2000 dataset.

### Model evaluation

We evaluate models with respect to how well they predict each fixation of a scanpath given the previous fixations made by a subject. We treat each fixation in a scanpath as a decision to saccade to a new image location, and we evaluate how well each of these decisions are predicted.

For a probabilistic model, this means evaluating the predicted *conditional fixation distribution* $p(x,y|x_{0},y_{0},\cdots ,x_{i-1},y_{i-1},I)$, for example, using metrics like average log-likelihood. For models that do not predict a probabilistic fixation distribution, instead we use the internal *priority map* (Kümmerer & Bethge, 2021): All included nonprobabilistic models build an internal model state over the previous ground truth fixations of the scanpath. In order to sample the next fixation, they construct a priority map encoding which image locations they consider good candidates for the next fixation and then apply, for example, winner-takes-all to select the next fixation. The main difference between conditional fixation distributions and conditional priority maps is that the latter do not have to be probability distributions, and fixations might be selected using a different strategy than probabilistic sampling from the distribution.

Both conditional fixation distributions and conditional priority maps can be evaluated using common saliency metrics. Here, we evaluate AUC (Judd et al., 2009) and NSS (Zhao & Koch, 2011). For probabilistic models, we can additionally report average log-likelihood relative to a uniform baseline model as “LL” and the information gain, which is average log-likelihood relative to the center bias baseline model, as “IG” (Kümmerer et al., 2015b). While IG, AUC, and NSS are commonly used to predict spatial fixation prediction models, simply by applying them to conditional predictions (and consequently evaluating each conditional prediction only on the one fixation that actually followed the given fixation history of the scanpath in question), they become sensitive to how well the model predicts the dependency between fixations in a scanpath. See Kümmerer and Bethge (2021) for an extensive summary of this evaluation method as well as details about how to apply it to existing models. We consider log-likelihood the most principled metric (Kümmerer et al., 2015b) and therefore use it as a main metric for all our internal analyses of DeepGaze III. For ranking all included models, which includes nonprobabilistic models and models trained on other datasets, we use the AUC metric. The AUC metric is only sensitive to the rank ordering of the prediction and therefore penalizes models least for not being optimized on the same dataset as we are using. Ideally, we would optimize and evaluate each model with maximum likelihood as in Kümmerer et al. (2015b), but this is much more computationally demanding for scanpath models than for static saliency models.

Many papers on scanpath modeling score the prediction quality of models by generating scanpaths from the model and comparing the generated scanpaths to human scanpaths using scanpath similarity metrics such as ScanMatch (Cristino et al., 2010) or MultiMatch (Jarodzka et al., 2010). However, these metrics can result in unreliable scores. For example, wrong models can score higher than the ground truth model even in simple and realistic cases. For this reason, we do not evaluate scanpath similarity metrics in the main paper. However, for comparison purposes, we include model scores for the scanpath similarity metrics ScanMatch and MultiMatch in the Appendix. For a much more extensive discussion of scanpath model comparison as applied here and the problems of scanpath similarity metrics for the purpose of model comparison, we refer to Kümmerer and Bethge (2021).

### Baseline models

To put the performance scores of models into perspective, we include several baseline models: The *uniform model* predicts fixations to be independently and uniformly distributed over the image. The *center bias model* quantifies how well fixations can be predicted without knowing the specific image that is being viewed and models the general tendency to look at the center of an image (Tatler, 2007). It is a Gaussian KDE model that uses the fixations of all other images to predict fixations on a given image and has an additional uniform regularization component. Its parameters are chosen for maximum likelihood on the MIT1003 dataset. The *spatial gold-standard model* is a mixture of a Gaussian KDE model that uses the fixations of all other subjects on the same image, the center bias model, and a uniform model. The underlying intuition is that fixations will usually be close to fixations of other subjects. But sometimes, especially due to the limited data per image, they will be better predicted by the general viewing tendency encoded in the center bias, and occasionally, subjects will make seemingly very random fixations. The parameters of the KDE and the mixture weights have been selected to reach maximum likelihood on the MIT1003 dataset with respect to the leave-one-subject-out cross-validation performance.

### Other models

Besides our baseline models, we also compare several other models of scanpath prediction. We included models that either reached high performance in our recent scanpath benchmark (Kümmerer & Bethge, 2021) or that are interesting in terms of their architecture, for example, by taking inspiration from neuroscience. We include the model of Itti and Koch (1998) using the implementation by Walther and Koch (2006); the STAR-FC model (Wloka et al., 2018); the MASC model (Adeli et al., 2017); the IOR-ROI-LSTM model (Sun et al., 2019); the SaltiNet model (Assens et al., 2017); the Saccadic Flow model (Clarke et al., 2017); the model of Le Meur and Coutrot (2016), which we refer to as “LeMeur16”; the SceneWalk model (Schütt et al., 2017), and its 2020 extension (Schwetlick et al., 2020), which we refer to as “SceneWalk20”; and the CLE model (Boccignone & Ferraro, 2004). Many scanpath models internally use a static saliency model to model the effect of scene content. From the above models, this is the case for MASC, LeMeur16, SceneWalk, SceneWalk20, and CLE. To make sure that these models do not suffer from using a worse saliency model than DeepGaze III, we use the scanpath-independent baseline version of DeepGaze III as their internal saliency model. Schwetlick et al. (2020) provided us with parameters for SceneWalk that have been fitted on MIT1003. For CLE, we optimized parameters on a subset of 100 images from MIT1003 for maximum likelihood. For all other models, we use the original parameters provided by the authors. See Kümmerer and Bethge (2021) for more details on implementation and parameters for each model.

## Results

### Prediction performance

#### MIT1003

In Table 2, we show prediction performance of DeepGaze III, the baseline models, and other included scanpath models on the MIT1003 dataset. Results are sorted by AUC since not all models allow evaluation of average log-likelihood. DeepGaze III reaches best prediction performance in all metrics. In log-likelihood, DeepGaze III scores 2.442 bit/fixation. Compared to the next best scanpath model in terms of log-likelihood (SceneWalk with 2.082 bit/fixation), DeepGaze III improves performance substantially by 0.360 bit/fixation. Interestingly, SceneWalk20 reaches lower performance than the original SceneWalk model. However, unlike the parameters used for SceneWalk, the parameters of SceneWalk20 are fitted on a different dataset than MIT1003. The low performance of IOR-ROI-LSTM in log-likelihood and information gain is also surprising given that the model was trained for maximum likelihood. However, the model applies a very strong saccadic prior after training, which results in overly confident model predictions. In the AUC metric, DeepGaze III reaches a score of 0.916 compared to 0.901 for the next best scoring scanpath model CLE. Finally, DeepGaze III reaches an NSS score of 3.257 compared to 2.699 for SceneWalk.

**Table 1.** Learning schedule. For each training phase, we indicate the used dataset, details of what was trained, initial learning rate, and the epochs after which the learning rate was decayed by a factor of 10. After the last stated epoch, training was stopped.

Training phase	Dataset	Details	Initial learning rate	Decay epochs
Phase 1	SALICON	Scanpath network removed	0.001	15, 30, 45, 60, 75
Phase 2	MIT1003	Scanpath network removed	0.001	3, 6, 9, 12, 15
Phase 3	MIT1003	First layer of spatial priority network fixed	0.001	10, 20, 30, 31, 32
Phase 4	MIT1003	All learnable parameters trained	0.00001	3, 6, 9

**Table 2.** Prediction performance on the MIT1003 dataset. Italic model names indicate baseline models; bold model names indicate the models presented in this article. Errors indicate standard deviation over eight repeated training runs. In all metrics, higher scores indicate better performance. Bold scores indicate best performance for that metric. Models are sorted by AUC.

Model	LL [bit/fix]	IG [bit/fix]	AUC	NSS
Itti&Koch (with WTA network)			0.473	0.271
*Uniform*	0.000	−0.906	0.500	0.000
STAR-FC			0.662	0.581
MASC			0.719	1.062
IOR-ROI-LSTM	−46.821	−47.727	0.744	0.457
SaltiNet	0.720	−0.186	0.790	1.138
*Center bias*	0.906	0.000	0.801	1.263
Saccadic Flow	1.170	0.264	0.843	1.603
LeMeur16	0.777	−0.128	0.862	2.336
**DeepGaze III w/o Spatial Priority**	1.695 ± 0.001	0.789 ± 0.001	0.874 ± 0.000	2.266 ± 0.004
SceneWalk20	1.852	0.947	0.885	2.683
**DeepGaze III w/o Scanpath**	1.945 ± 0.003	1.039 ± 0.003	0.889 ± 0.000	2.582 ± 0.004
SceneWalk	2.082	1.176	0.900	2.699
CLE	1.841	0.935	0.901	1.437
*Spatial Gold Standard*	2.120	1.215	0.901	2.853
**DeepGaze III**	**2.442 ± 0.010**	**1.536 ± 0.010**	**0.916 ± 0.001**	**3.257 ± 0.016**

Except for the uniform and the center bias baseline models, the Saccadic Flow model and the version of DeepGaze III without the spatial priority network (“DeepGaze III w/o spatial priority”) are the only image-independent models in our evaluation. Here, the image-independent version of DeepGaze III improves the log-likelihood by 0.525 from 1.170 bit/fixation to 1.695 bit/fixation (however, the Saccadic Flow model has access only to the latest fixation location). We discuss the different ablated models in more detail below.

#### MIT300

In Table 3, we show the performance of DeepGaze III on the MIT300 holdout dataset from the MIT/Tuebingen Saliency Benchmark (Judd et al., 2012; Kümmerer et al., 2018) compared to other state-of-the-art models. Model scores of other models are as reported by Kümmerer and Bethge (2021). As on MIT1003, DeepGaze III sets a new state-of-the-art on MIT300 in all metrics. Compared to the best other model (SceneWalk using DeepGaze II as saliency model), DeepGaze III increases average log-likelihood by 0.325 bit/fixation from 1.923 bit/fixation to 2.248 bit/fixation and AUC by 0.016 from 0.890 to 0.906. The performance gain compared to previous models is very similar on MIT1003 and on MIT300, indicating that DeepGaze III is not overfit to MIT1003 in training.

**Table 3.** Prediction performance on the MIT300 (holdout) dataset of the MIT/Tuebingen Saliency Benchmark. For comparison, we include the top-performing models as evaluated on MIT300 in Kümmerer and Bethge (2021). Where applicable, we indicate in parentheses which static saliency model a scanpath model used (DG2=DeepGaze II) and whether original model parameters were finetuned for MIT1003. In all metrics, higher scores indicate better performance. Bold scores indicate best performance for that metric. Models are sorted by AUC.

Model	LL [bit/fix]	IG [bit/fix]	AUC	NSS
Saccadic Flow	1.070	0.287	0.835	1.491
LeMeur16 (DG2)	0.581	−0.201	0.850	2.123
DeepGaze II	1.724	0.942	0.873	2.337
CLE (DG2, finetuned)	1.686	0.904	0.888	1.490
SceneWalk (DG2, finetuned)	1.923	1.141	0.890	2.530
**DeepGaze III**	**2.248**	**1.466**	**0.906**	**2.957**

#### CAT2000

In Table 4, we report model performance on the CAT2000 dataset. Model scores of other models are as reported by Kümmerer and Bethge (2021). DeepGaze III sets a new state of the art in all reported metrics with a log-likelihood of 3.064 bit/fixation, AUC of 0.932, and NSS of 5.106. The next best other model in all metrics is the CLE model (LL = 2.581 bit/fix, AUC = 0.915, NSS = 3.453). Compared to this model, DeepGaze III increases log-likelihood by 0.483 bit/fixation, AUC by 0.017, and NSS by 1.653. While especially the performance gains in log-likelihood and NSS are larger than on MIT1003, this should not be overinterpreted. On MIT1003, we could compare to some models fitted on the same dataset, while this is not the case for CAT2000: The SceneWalk model, which was the best model except for DeepGaze III on MIT1003 and MIT300, drops by several ranks on CAT2000 (LL = 1.806 bit/fix, AUC = 0.853, NSS = 2.708). On MIT1003 and MIT300, we could use model parameters trained on MIT1003 for SceneWalk, but for CAT2000, we had to resort to the published model parameters, which have been fitted on a different dataset. This most likely explains the drop in performance of SceneWalk. The image-independent version of DeepGaze III (“DeepGaze III w/o spatial priority”) improves the log-likelihood compared to the image-independent Saccadic Flow model by 1.159 from 1.721 bit/fixation to 2.880 bit/fixation.

**Table 4.** Prediction performance on the CAT2000 dataset. Italic model names indicate baseline models; bold model names indicate the models presented in this article. Performance scores of other models are as evaluated on CAT2000 in Kümmerer and Bethge (2021). Where applicable, we indicate in parentheses which static saliency model a scanpath model used (DG2=DeepGaze II). In all metrics, higher scores indicate better performance. Bold scores indicate best performance for that metric. Models are sorted by AUC.

Model	LL [bit/fix]	IG [bit/fix]	AUC	NSS
Itti&Koch (with WTA network)			0.379	−0.003
*Uniform*	0.000	−1.439	0.500	0.000
STAR-FC			0.610	0.303
MASC (DG2)			0.676	0.835
IOR-ROI-LSTM	−53.386	−54.825	0.687	0.222
SaltiNet	0.918	−0.521	0.823	1.329
SceneWalk20 (DG2)	1.412	−0.027	0.844	2.806
LeMeur16 (DG2)	−0.700	−2.139	0.844	1.711
*Center bias*	1.439	0.000	0.849	2.156
SceneWalk (DG2)	1.806	0.367	0.853	2.708
**DeepGaze III w/o Scanpath**	1.803	0.364	0.880	2.420
*Spatial Gold Standard*	1.882	0.443	0.885	2.517
Saccadic Flow	1.721	0.282	0.907	2.199
CLE (DG2)	2.581	1.142	0.915	3.453
**DeepGaze III w/o Spatial Priority**	2.880	1.441	0.922	4.975
**DeepGaze III**	**3.064**	**1.625**	**0.932**	**5.106**

### Scanpath statistics

**Figure 3.**
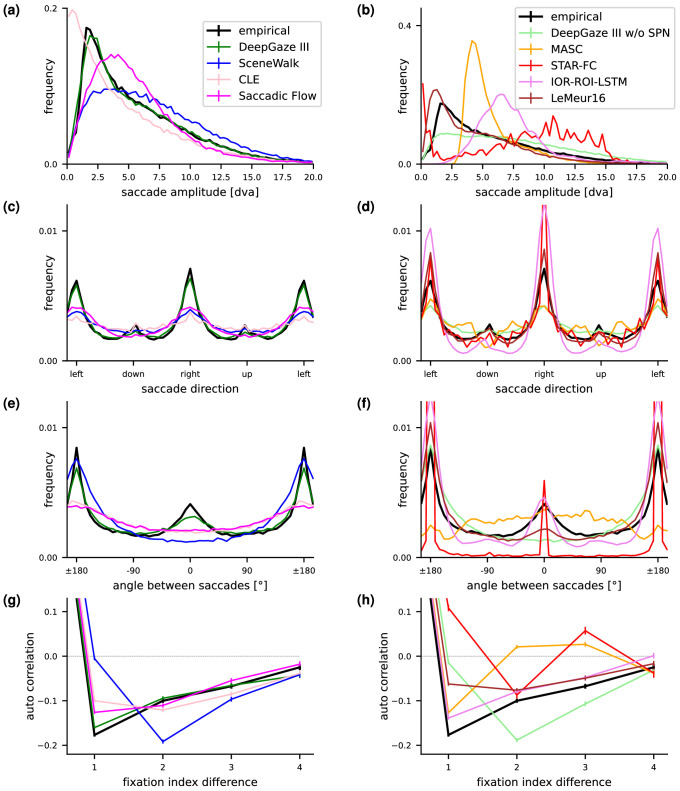
We inspect how well different models reproduce several statistical properties of human scanpaths. For better visibility, we distribute models over two subplots with the top-performing scanpath models on the left. (a, b) Saccade amplitude. (c, d) Saccade direction. (e, f) Angle between saccades. (g, h) Autocorrelation between saccade amplitudes in scanpaths. DeepGaze III w/o SPN = DeepGaze III without scanpath network. Error bars indicate bootstrapped 95% confidence intervals for the mean. In subplots a to f, they are so small that they would be invisible or barely visible.

Human scanpaths exhibit a range of well-known key statistical properties, such as the distribution of saccade amplitudes, tendencies toward horizontal and vertical saccades, and dependencies between consecutive saccade directions (see, e.g., Tatler & Vincent, 2008, 2009; Smith & Henderson, 2009; Wilming et al., 2013; Rothkegel et al., 2016). In order to check how well DeepGaze III reproduces these statistical properties, for each scanpath of our ground truth human scanpath data, we sampled a new scanpath from DeepGaze III on the same image, starting with the same initial fixation and up to the same length. The same process was repeated for the models CLE, IOR-ROI-LSTM, LeMeur16, MASC, Saccadic Flow, SceneWalk, and STAR-FC. Since some of the statistical properties such as saccade direction could be partially explained purely by the spatial distributions of salient objects in the image, we also included our spatial baseline model (DeepGaze III without scanpath network). We then compared how well the sampled scanpaths matched the ground truth data with respect to the distribution of saccade amplitudes, saccade directions, angle between saccades, and autocorrelation between saccade amplitudes. The results are shown in Figure 3.

In Figures 3a,b, we show the distribution of *saccade amplitudes*. The empirical data show a strong peak at around 2 degree of visual angle (dva) and then slowly decays until around 20 dva. DeepGaze III matches this distribution nearly perfectly. The CLE model has a quite similar distribution, but due to the nature of the Levy flight, the peak is close to zero. The SceneWalk model matches the heavy tail quite well but fails to reproduce the sharp peak. Most other models have either a tendency toward too short or too long saccades.

In Figures 3c,d, we show the distribution of *saccade directions*. The empirical data show a strong tendency toward horizontal and also to some degree toward vertical saccades. DeepGaze III reproduces the trend toward horizontal saccades quite well, but the tendency toward vertical saccades is a bit too small. Already, the purely spatial version of DeepGaze III shows the effects to a certain extent due to the alignment of salient objects in images. The IOR-ROI-LSTM model reproduces the qualitative distribution quite well since empirical saccade direction biases are an explicit part of the model. However, quantitatively, the effects are too strong. The SceneWalk model shows the effects, but the horizontal peaks are smaller and wider.

In Figures 3e,f, we show the distribution of *angles between saccades*. The empirical data show a strong tendency toward angles of $0^{\circ }$ and $180^{\circ }$, corresponding to either making two saccades in the same or in opposite directions. DeepGaze III reproduces both peaks, especially the one at $180^{\circ }$. The peak at $0^{\circ }$ is not strong enough. SceneWalk and CLE reproduce only the effect of antiparallel saccades. IOR-ROI-LSTM and STAR-FC reproduce the effects qualitatively, but much too strong.

In Figures 3g,h, we show the *autocorrelation between saccade amplitudes in scanpaths*. The empirical data show that the amplitude of two consecutive saccades is anti-correlated with a correlation coefficient of approximately −0.2 and that this correlation slowly decays for later saccades. This shows that long saccades are often followed by short saccades and vice versa. DeepGaze III and IOR-ROI-LSTM reproduce this effect very precisely. CLE shows the effect nearly as well. DeepGaze III without scanpath network does not show the anticorrelation of consecutive saccade amplitudes, suggesting that the placement of salient objects is not enough to explain the effect.

**Figure 4.**
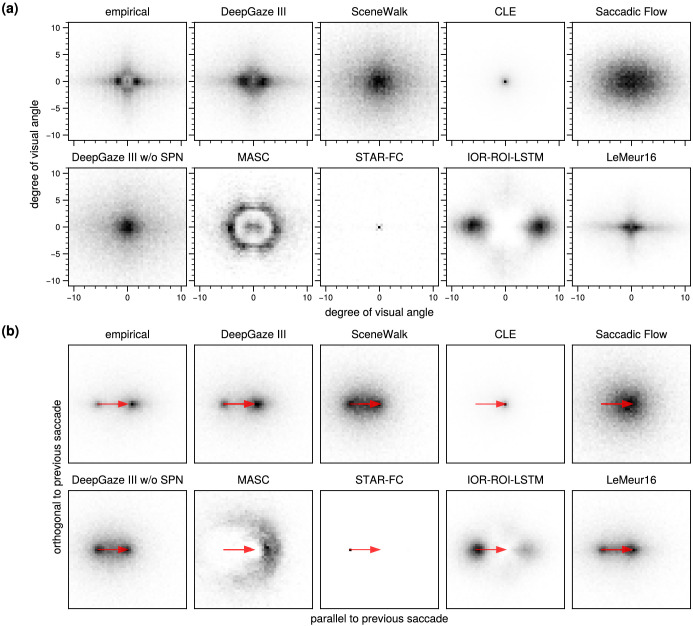
(a) Distribution of saccades in x and y direction for human scanpath data (empirical) and data sampled from scanpath models. (b) Distribution of next fixation relative to last saccade (red arrow). DeepGaze III w/o SPN = DeepGaze III without scanpath network.

In Figure 4a, we show the two-dimensional distribution over saccade vectors. The empirical data show a strong tendency toward horizontal saccades with an amplitude of about 2 dva. DeepGaze III reproduces this effect, but the two peaks for left and right saccades are less peaked. The distribution for CLE is very peaked due to the nature of the underlying Levy flight, where saccade amplitudes follow a Cauchy distribution. The distribution for STAR-FC is also very peaked since the model usually produces a very short saccade after each longer saccade. IOR-ROI-LSTM shows two pronounced peaks for left and right saccades, but they correspond to saccade amplitudes of approximately 6 dva compared to around 2 dva in the empirical data. LeMeur16 captures the tendency toward horizontal and vertical saccades quite well, but shows a tendency toward too short saccades.

Finally, we checked how well DeepGaze III and other models capture the dependency between consecutive saccades with respect to saccade amplitude and saccade angle (Figure 4b; Rothkegel et al., 2016). We rescaled and rotated all saccades such that the previous saccade is a rightward saccade of unit length (red arrow) and visualized the distribution of resulting saccade landing points (heatmap). The empirical data show that saccades mostly go roughly in the same direction as the previous saccade but have a much shorter amplitude. These saccades could be either saccades to a close-by area of the same object after a long saccade from another object to this object, or they could be corrective saccades (Becker & Fuchs, 1969; Bahill & Troost, 1979; Lisi et al., 2019). There is, however, also a very pronounced effect of return saccades: A substantial number of saccades go very precisely back to the location where the previous saccade started (Wilming et al., 2013). DeepGaze III reproduces this distribution quite well, although both the return saccade effect and the effect of shorter saccades in a similar direction are more scattered than in the empirical data. SceneWalk shows only a subtle effect of shorter saccades in a similar direction but has an even too strong tendency to return to the previous fixation position (which might be mainly saliency driven, since the scanpath-independent baseline model shows the same effect). For STAR-FC, we see a very strong peak a the end of the last saccade. This is again because STAR-FC tends to make an extremely short saccade after each longer saccade. LeMeur16 seems to capture the distribution nearly as well as DeepGaze III, but the tendency to make return saccades seems to be too strong and additionally there is a tendency toward making saccades in the return direction but of smaller amplitude than the last saccade.

### Qualitative analysis

**Figure 5.**
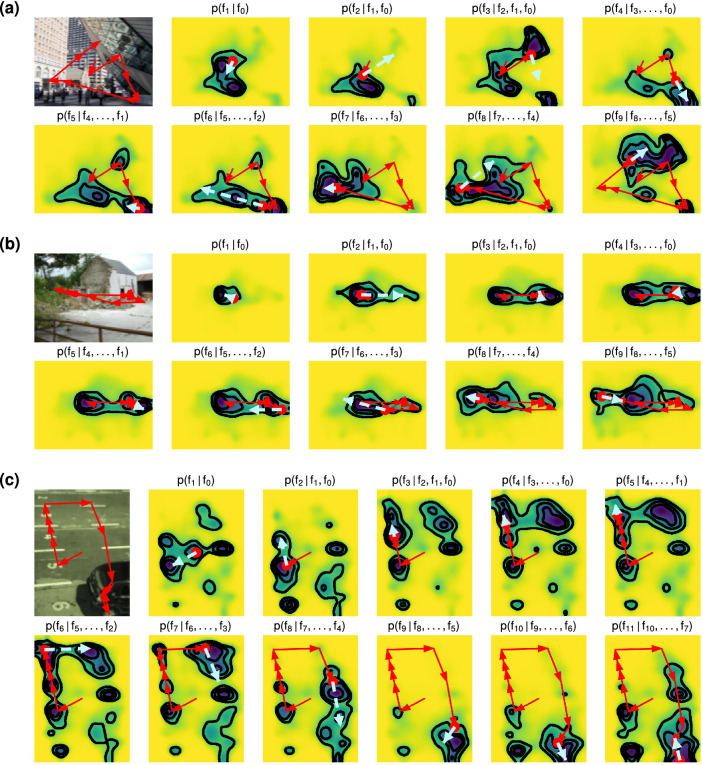
Model predictions for example scanpaths. For three scanpaths (a, b, c), we show model predictions. In each subpanel, the first image shows the viewed image with the scanpath overlaid. Subsequent plots left to right show model predictions for the first free fixation, the second free fixation, and so on. In each prediction, we show the scanpath history so far (red arrows), the current gaze position (red circle), and the following saccade (cyan dashed arrow), which the model is supposed to predict. Contour lines visualize the four quantiles with 25% probability mass each.

In Figure 5, we show model predictions for three example scanpaths. For each scanpath, we first show the viewed image with the subject's scanpath superimposed. The subsequent plots show for each fixation of the scanpath the model predictions and are superimposed with the scanpath history so far (red arrows) and the next saccade that the model has to predict (cyan arrow).

The figure shows that that model predictions strongly depend on the previous scanpath history: DeepGaze III has learned it is useful to take the previous scanpath history into account to make more precise predictions. DeepGaze III predicts that usually the next fixation will be not too far from the current fixation; however, it considers long saccades to high-level objects or otherwise interesting content still as possible. Furthermore, the predictions overall appear quite correct as far as it can be judged from those few samples: In most cases, the next fixation is within the nonyellow area, which visualizes the pixels with highest probability, which sum up to a total probability mass of 75% and therefore should contain 75% of all fixations.

**Figure 6.**
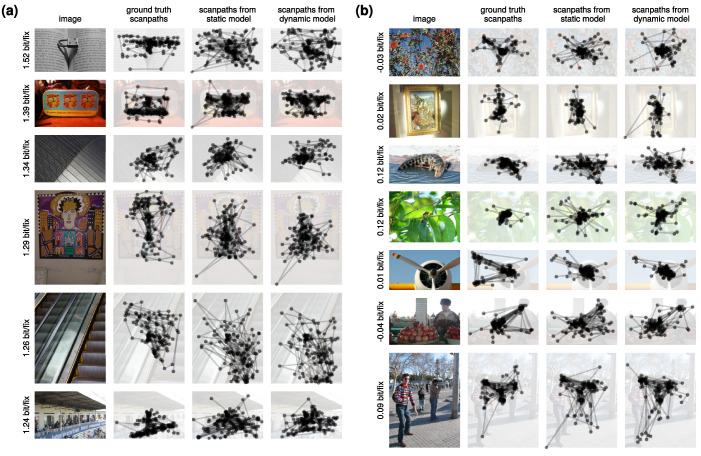
Case studies. (a) Images where DeepGaze III's log-likelihood most improves over the static baseline model. Qualitatively, scanpath information most improves predictions in capturing the distribution of short saccades, particularly when text is present. (b) Images where the static baseline model has a performance similar to DeepGaze III. Scanpath information seems to be less predictive when scenes are either landscapes or contain multiple small salient objects, where observers make long saccades between these objects. Note that both models miss significant scanpath structure in some images, indicating there is still room for improving DeepGaze III. Numbers to the left indicate the log-likelihood difference between DeepGaze III and the static baseline model. The three columns next to each image show the ground truth scanpaths, scanpaths that have been sampled from the static baseline model, and scanpaths that have been sampled from DeepGaze III.

Finally, we present “case studies” of images for which scanpath history matters most or least compared to static saliency. Specifically, we hand-selected qualitatively interesting images from the top and bottom 20 images where DeepGaze profits most or least from having access to the scanpath history, by comparing the prediction performance of our static baseline model and the full DeepGaze III model on individual images. In Figure 6a, we show some of the images where DeepGaze III's log-likelihood improves most over the static baseline model. Several of these contain many short saccades (e.g., due to subjects reading text). The static baseline model cannot capture this effect, resulting in substantially worse performance than DeepGaze III. In Figure 6b, we show some of the images where DeepGaze III's log-likelihood is worst compared to the static baseline model. Scanpath information seems to be less predictive when scenes either contain multiple small salient objects, where observers make long saccades between these objects, or when they contain just one salient object, resulting in mostly extremely short saccades. However, the scanpaths sampled from the models indicate that for these images, both models still miss parts of the scanpath structure. The missing patterns seem mainly to be due to missing salient objects such as the most relevant text in an image, but partially also due to missing patterns in the sequence of fixations. DeepGaze III also seems to be underconfident, assigning too much likelihood to the background. Although visual comparisons of scanpaths on single images should not be overinterpreted (Kümmerer & Bethge, 2021), these examples show that there is still room for improving DeepGaze III.

### Effect of image content and scanpath dependency order

**Figure 7.**
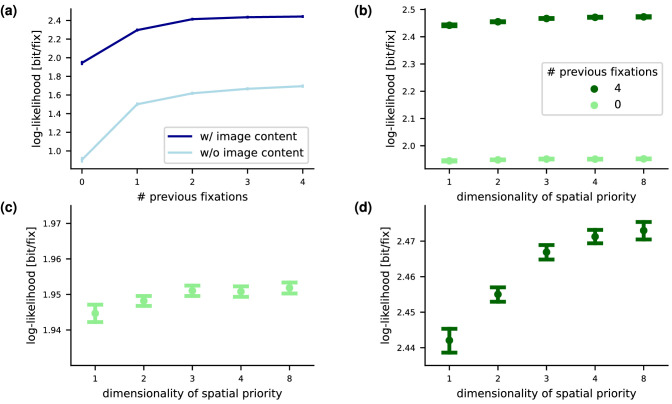
Effect of image content and scanpath dependency order on prediction performance on the MIT1003 dataset. (a) We trained versions of DeepGaze III that either have access to image content via the DenseNet backbone or do not have access to image content and that have access to zero, one, two, three, or four previous fixations. We show prediction performance in average log-likelihood. (b) By increasing the dimensionality of the internal spatial priority map from one to higher, DeepGaze III can capture nontrivial interactions between scene content and scanpath history. We show prediction performance for models with different dimensionalities of the spatial priority map for both the purely spatial baseline (light green) and the full scanpath model (dark green). (c) Zoomed-in view of the performances of the spatial baseline models. (d) Zoomed-in view of the performances of the full scanpath models. All error bars are bootstrapped 95% confidence intervals for the mean log-likelihood per image using the normalization method of Cousineau (2005) for paired comparisons with the correction of Morey (2008).

DeepGaze III combines information about the image content with information about up to four previous fixations. In order to assess how relevant these different kinds of information are, we trained versions of the model without access to part of the information. We removed the information about image content by replacing the output of DenseNet with a single constant feature map. We removed the information about previous fixations by feeding fewer feature maps to the scanpath network (only information about the last three or two fixations or only about the last fixation) or by removing the scanpath network altogether (essentially converting the model into DeepGaze II). In the extreme case (no image information, no scanpath information), the model reduces simply to the center bias. In Figure 7a, we show how much these ablations affected prediction performance. Having access to image content in a static model improves performance from 0.91 bit/fix by 1.04 bit/fix to 1.94 bit/fix. Furthermore, adding information about the scanpath history with up to four previous fixations increases performance by 0.50 bit/fix to 2.44 bit/fix. On the other hand, adding scanpath information to the center bias model increases performance by 0.79 bit/fix to 1.69 bit/fix. When having access to image content, most additional information about scanpath history is added by the current fixation (0.35 bit/fix). The second to last fixation adds with 0.12 bit/fix already much less information, and the contribution of the earlier fixations is hardly measurable (0.02 bit/fix and 0.01 bit/fix)

When not having access to image content, each additional previous fixation adds more information and even the third to last and fourth to last fixations still add some information (0.05 bit/fix and 0.03 bit/fix). However, this is to be expected: In the absence of image information, previous fixations can be used to approximate the missing spatial priority map and therefore improve the prediction performance.

The fact that prediction performance of the image content-aware model does not increase after two previous fixations suggests that saccades returning to a previous fixation location that was not fixated directly before are not driven by fixation history but only by spatial priority.

Taken together, these results indicate that under the experimental conditions of the MIT1003 dataset, scene content has a stronger effect on fixation selection than previous scanpath history (though both are important). Furthermore, the current fixation position seems to have a much stronger effect than the previous fixation position, and earlier fixation positions have next to no influence. The strong effect of the current fixation position might be expected since some of the most prominent scanpath properties, such as saccade amplitude and direction distributions, depend only on the current fixation position. The next to inexistent effect of fixations earlier than last two fixations is more interesting: It shows that effects like (spatial) inhibition of return or excitation of return are already completely decayed after two fixations. This is in contrast to how inhibition of return is handled in many scanpath models (e.g., Itti et al., 1998; Adeli et al., 2017; Wloka et al., 2018; Xia et al., 2019).

**Figure 8.**
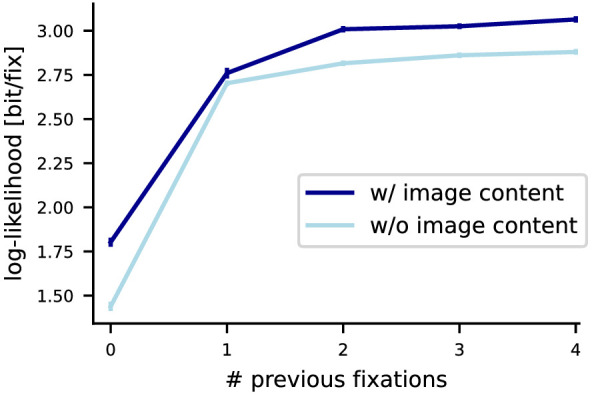
Effect of image content and scanpath dependency order on prediction performance on the CAT2000 dataset. We trained versions of DeepGaze III that either have access to image content via the DenseNet backbone or do not have access to image content and that have access to zero, one, two, three, or four previous fixations. We show prediction performance in average log-likelihood. All error bars are bootstrapped 95% confidence intervals for the mean log-likelihood per image using the normalization method of Cousineau (2005) for paired comparisons with the correction of Morey (2008).

We also evaluated model performance as a function of dependency order on the CAT2000 dataset (Figure 8). As in MIT1003, we again see that the last two fixations are most relevant in terms of scanpath dynamics. However, unlike on MIT1003, the effect of image content is much smaller (0.36 bit/fix from the static image-independent model to the static image-dependent model) than the effect of scanpath history (1.44 bit/fix from the static image-independent model to the image-independent model of order 4).

There are a number of likely reasons for these differences: Compared to the natural scenes in MIT1003, some categories of CAT2000 either have many salient areas (satellite images) or might be visually not very informative (fractal, noisy, pattern, low resolution). In these cases we expect image content to constrain fixation placement less strongly than on MIT1003. This expectation is confirmed when comparing model performances per category (see Appendix, Table 7): The performance difference between the center bias and the model without access to scanpath history (i.e., only with access to image content) for these categories is less than 0.2 bit/fix, while categories closer to the MIT1003 dataset (e.g., Outdoor, Social, Action) show values up to 0.6 bit/fix. However, even this is still substantially smaller than in the MIT1003 dataset.

Differences in the experimental setup are the most likely explanation for the remaining differences: the stimuli in CAT2000 are substantially larger (diagonal of about 55 dva) than the stimuli in MIT1003 (diagonal about 35 dva). Therefore, constraints such as saccade length distribution restrict possible saccade landing locations much more than in MIT1003, resulting in a higher contribution of scanpath history. Additionally, presentation time in CAT2000 is longer than in MIT1003, and subjects might have enough time to also explore image areas with less interesting content. The larger stimulus size is also the likely source for the much stronger center bias in the CAT2000 dataset (1.44 bit/fix compared to 0.91 bit/fix for MIT1003). The relatively small gap between the model performances for order 1 in Figure 8 is most likely a result of training noise. Unlike for MIT1003, we could not afford to train each model multiple times on the much larger CAT2000 dataset.

### Interactions between scene content and scanpath history

Most scanpath models assume the existence of some kind of saliency map that encodes the relevance of each image region into a single scalar value. This saliency information is then combined with information about the previous scanpath history (e.g., via inhibition of return) to select future fixation locations. The model architecture of DeepGaze III also makes this assumption, because all image information is encoded into a single spatial priority map before combining the spatial priority map with the scanpath history (see Figure 2). However, this is a strong assumption, because the model cannot capture nontrivial interactions between scene content and scanpath history. For example, consider the hypothetical possibility that simpler image features (luminance and color contrast) are more important for determining the next eye movement after a long saccade is completed, but complex image features (objects) are more important after short saccades. Capturing this dependency is impossible in the single spatial priority map version of DeepGaze III, because the image features are inaccessible to the scanpath network behind the single-channel bottleneck and cannot be reweighted.

We can test whether the MIT1003 dataset provides evidence for such complex interactions by relaxing this bottleneck assumption in DeepGaze III. If we give the internal spatial priority map multiple channels, the model can compute multiple spatial priority maps and then use the scanpath history to combine them. This would allow the model to capture nontrivial interactions such as the one described above. We therefore trained multiple versions of DeepGaze III where the internal spatial priority map is of different dimensionality, from the standard single-channel spatial priority map up to eight channels. Except for this difference, the model architecture and training paradigm were identical.

In Figures 7b–d, we show results for this experiment. Figure 7b shows that the overall model performance is close to 2.45 bit/fix independent of the dimensionality of the spatial priority map, compared to 1.95 bit/fix for the scanpath-independent version of the model. In Figure 7c, we zoom into the performances of the scanpath-independent models and see that the log-likelihood remains essentially flat from spatial priority of dimensionality 2 and higher, and all change is very small in scale, less than 0.01 bit/fix. This is an important control condition: If performances would clearly increase with spatial priority dimensionality for the scanpath-independent models, this would suggest that the spatial priority network is not powerful enough, because with higher spatial priority dimensionality, image information leaks into the fixation selection network where it can be used to improve the final spatial priority map. In Figure 7d, we zoom into the performances of the scanpath-dependent models. Here, the results appear quite different: There is a clear increase in performance with increasing spatial priority dimensionality. This indicates there are indeed some nontrivial interactions between scene content and scanpath history. However, as can be seen in Figure 7b, these interactions have only marginal effects on fixation selection compared to the simple, spatial priority-based effect that we already see with a one-dimensional spatial priority map.

### Identifying different contributions to the center bias

**Figure 9.**
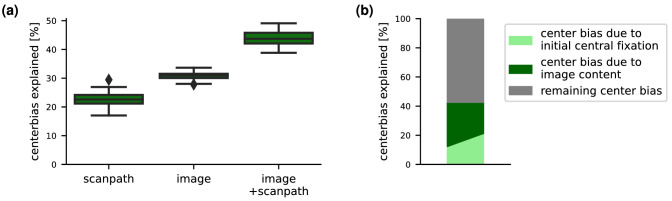
Decomposing the center bias: DeepGaze III has access to the overall center bias of MIT1003. Part of the center bias can be explained by photographer bias as well as by the combination of initial central fixation and limited presentation time; therefore, the model can learn to downweight the overall center bias. (a) For models with access to scanpath history, image content, or both, we show which percentage of the center bias is explained away. Error bars indicate the distribution over eight training runs. (b) From the percentage of center bias explained away by the different models, we can decompose the overall center bias of the dataset into contributions from the initial central fixation, from the image content and a remaining bias to fixate closer to the center of the image independent of image content and scanpath history. Some part of the center bias can be explained by both image content and scanpath history, which is why the separation line between them is not horizontal.

There is a well-known tendency to fixate closer to the center of an image, the center bias. The center bias can be partially explained by the fact that photographers often tend to put objects in the center of the image (*photographer bias*). Besides that, depending on the dataset, part of the center bias can stem from a combination of the initial central fixation and a limited presentation time: Since we tend to make short saccades, early fixations will be close to the image center. However, it is known that these factors do not explain the observed center bias to its full extent (Tatler, 2007). The architecture of DeepGaze III allows us to disentangle the observed center bias into different contributions. DeepGaze III combines the output of the readout networks with the provided overall center bias but can learn to downweight the center bias contribution. Therefore, it can account for the fact that it might be able to explain part of the center bias from the image content or the previous fixations. By comparing the learned center bias weights for different instances of DeepGaze III that have access to scanpath history, image content, or both, we can quantify how much photographer bias and initial central fixation contribute to the center bias in our dataset. The results are visualized in Figure 9a. We find that the dependency on previous fixations explains 22.6%±2.1% of the overall center bias. The image content on its own explains 30.8%±1.1%. Scanpath history and image content together explain 43.8%±2.5% of the full center bias. This number is smaller than the sum of the individual percentages for image content and scanpath history, respectively: Apparently, the parts of the center bias explained by the two individual models are not completely disjoint. A part of 9.6% (22.6%+30.8%-43.8%) of the center bias can be explained both by image content or scanpath history; our data are inconclusive on this point. This is likely at least partially due to the fact that the scanpath history also contains information about the salient image regions and therefore about the image-driven center bias. In this case, image content would be the actual source of that part of the center bias. Finally, about 60% of the overall center bias cannot be explained by image content or the initial central fixation but seems to reflect purely a preference to fixate closer to the center of the image (or screen). This analysis of different contributions to the center bias is visualized in Figure 9b.

## Predictability over time

DeepGaze III assumes that the placement of the next fixation only depends on a limited number of previous fixations and that this dependency does not change over the course of a scanpath. Obviously, this is a simplification that will not hold perfectly.

**Figure 10.**
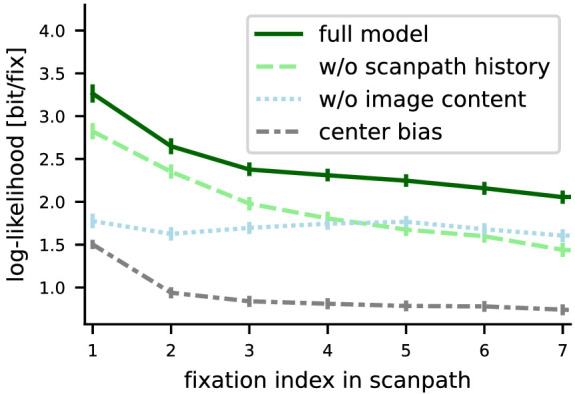
Prediction performance of MIT1003 depending on fixation index, starting with the first free fixation. We show prediction performance as log-likelihood in bit/fixation as a function of the index of fixations in the scanpath, for the full DeepGaze III model as well as ablated models without access to scanpath history, image content, or both (i.e., the center bias). The results suggest that subjects scan central-most salient areas in the first fixation, then the most salient areas all over the image in the second fixation, and then scan increasingly less salient areas while at the same time becoming slightly more predictable in terms of scanpath dynamics. The part of the central fixation bias that is not dependent on image content seems to stay constant over the course of the scanpath (see main text). Error bars are bootstrapped 95% confidence intervals for the mean log-likelihood per image using the normalization method of Cousineau (2005) for paired comparisons with the correction of Morey (2008).

In Figure 10, we show prediction performance in log-likelihood as a dependency of how early or late the predicted fixation occurs in a scanpath. For the full DeepGaze III model, log-likelihood decays substantially until the third fixation, after which the decays continues much slower. This means that earlier fixations can be better predicted than later fixations. Multiple reasons could explain this effect: First, it might be that earlier in the viewing progress, we first scan only the most salient objects. Second, it could be that over the course of the scanpath, the dynamics of the scanpath change (e.g., that we start to make longer saccades). Finally, it is well known that the observed center bias decays over time (e.g., Koehler et al., 2014). However, it is not clear whether this also applies to “remaining center bias” (see previous section), that is, the part of the center bias that is not explained by image content or scanpath dynamics and the initial central fixation.

To distinguish between these possibilities, we also look at the performance of the ablated models, in which we remove access to scanpath history, image content, or both (Figure 10, nonsolid lines). We find that both the center bias model and the scanpath-independent model show a similar decay in log-likelihood as the full model. However, the model without access to image content shows relatively constant performance over time. This suggests that neither the dynamics of the scanpath nor the “remaining” or dynamic dependent part of the center bias change substantially over time. Therefore, the decay in prediction performance in the other three models has to be explained by image content: In the first fixation, subjects seem to scan high-salience image areas that are also close to the image center (hence the high performance of the center bias model and both image-dependent models). In the second fixation, subjects still seem to scan very high-salience images, but they can be anywhere in the image (the image-dependent models still have high performance, but the center bias model already decayed mostly to the performance for the remaining fixations). From the third fixation on, the full model still decreases slowly in performance, but slower than the scanpath history-dependent model. This suggests that now subjects start to look at increasingly less salient image areas, but at the same time, the dynamics of the scanpath become more predictable. The last hypothesis is also supported by the fact that the image-independent model gains slightly in performance starting with the third fixation: Subjects seem to deviate from the usual scanpath dynamics in the first two fixations in order to reach high-salience image areas.

Taken together, we find that observers seem to explore the image in two phases. There is an initial phase of two to three fixations where high-saliency objects are scanned, starting with those close to the image center and then all over the image. In this phase, image content is substantially more important than scanpath history. After the initial phase, subjects look at increasingly lower-saliency image regions and the saccades are now more driven by the scanpath history. One might expect that after the first few fixations, scanpath history becomes more relevant than image content, simply because there is more scanpath history to use. However, interestingly, the more prominent effect is the decrease of the influence of image content, while the effect of scanpath history stays close to constant.

These results confirm and extend an earlier analysis by Schütt et al. (2019), which focused only on the spatial fixation distribution without taking scanpath history into account.

## Discussion

We presented DeepGaze III, a new deep learning–based model for predicting human scanpaths when free-viewing natural images. DeepGaze III combines information about image content produced by deep neural networks trained on object recognition with information about previous fixations a subject made to predict where the subject might fixate next. Trained on human scanpath data, DeepGaze III sets a new state of the art, explaining 2.442 bit/fixation compared to a uniform baseline model on the MIT1003 dataset, 2.248 bit/fixation on the MIT300 dataset, and 3.064 bit/fixation on the CAT2000 dataset. Besides achieving high prediction performance, DeepGaze III also captures many key statistical properties of human scanpaths such as a tendency toward horizontal and vertical saccades or a tendency to make return saccades.

While DeepGaze III is a deep learning model that is optimized for prediction performance, it uses a modular architecture consisting of a spatial priority network, a scanpath network, a fixation selection network, and a center bias. This allowed us to conduct several ablation studies and quantify the relevance of different parts of the input data. For example, in our main dataset, scene content has a stronger effect on fixation selection prediction than previous scanpath history (though both are important). In addition, the effect of scanpath history in free-viewing tasks comes mostly from the current fixation position and only to a much smaller part from the last fixation position.

We suggest that these kinds of conclusions would be difficult to draw from classic mechanistic models. Because these models are comparatively highly capacity limited and often inflexible, it is difficult to draw conclusions about the overall importance of, for example, scene content and scanpath history from model performances or ablations alone. Is poor predictive performance because those sources of information or behavior are unimportant, or because the particular mechanism instantiated in the model is the wrong way to capture that information? On the other hand, neural networks are universal function approximators. This means that they can extract all complex and hidden patterns from the data (if the model is complex enough) and therefore estimate how well scanpaths can be predicted, for example, from scene content, scanpath history, or both, in a given dataset.

Therefore, we argue that deep learning allows models like DeepGaze III to stand in as a proxy for the empirical densities or gold-standard models that are used in spatial fixation prediction to estimate the achievable performance (Kümmerer et al., 2015b). Since scanpaths are high-dimensional, the empirical scanpath distribution for a given image cannot be estimated using, for example, a KDE without immense amounts of data. Here, DeepGaze III provides a lower (but reasonably high) bound on the amount of explainable information in human free-viewing scanpaths. This allows one to put the prediction performance of mechanistic models in perspective and quantify how relevant the proposed mechanisms are for predicting the fixation selection process as a whole. The mechanisms proposed by models such as SceneWalk, which are founded in results from neuroscience and psychophysics, are able to account for about 28% of the information gain between spatial baseline model and full model. Given that DeepGaze III is only a lower bound on the explainable information gain, this suggests that there are substantial additional effects at play that are so far not fully understood (see also the failures to capture scanpath structure in Figure 6b).

Our analysis of the possible interactions between scene content and scanpath history provides another example of the contributions that deep learning can provide for vision science. Many existing scanpath models strongly constrain the possible interactions between scene content and scanpath history by first computing a saliency map from the image, which is then used to select the next fixation position (e.g., SceneWalk, MASC, and CLE), whereas some models do not implement a saliency map at all (e.g., IOR-ROI-LSTM). However, these models differ in many additional aspects, making it difficult to draw general conclusions about the value of a saliency map. We were able to show that, at least for the analyzed dataset, most of the predictive power of interactions between scene content and scanpath history can be captured by a single scalar saliency (or, as we call it, spatial priority) measure. This provides support for the hypothesis of a unified scalar saliency map for guiding gaze position, at least under free-viewing conditions. This is not to say that a single saliency map or priority map, which integrates all ways in which scene content affects fixation selection, must be represented in the brain. Under more naturalistic or task-driven viewing conditions, we very well expect there to be complex interactions between scanpath history and scene content (for example, in sequential searching for single or multiple targets; Hoppe & Rothkopf, 2019; Wolfe, 2021). Even for free-viewing conditions, we see some subtle but measurable effects that cannot be explained by a single spatial priority map (Figure 7d). We leave exploring them in more detail for future work. For the presented analysis, it is important that we can assume that our different models pick up on all relevant structure in the data except for the constraints that we intentionally build into the models, such as a single-channel spatial priority map. This is exactly what deep learning methods can provide.

Another implication from our single- versus multichannel spatial priority analysis concerns the difference between retinotopic and spatiotopic saliency. Some models (e.g., STAR-FC; Wloka et al., 2018) implement saliency maps as retinotopic, in the sense that different image features determine what is salient depending on the distance to the fovea. In the DeepGaze III architecture, modeling this type of dependency would require multiple spatial priority map channels. Each spatial priority channel would compute a spatial priority value for a certain distance to the fovea. The fixation selection network has access to the distance to the last fixation and could use it to select the corresponding spatial priority channel for each pixel, creating a retinotopic spatial priority prediction over the whole image. The fact that multiple spatial priority channels do not perform substantially better than a single-channel spatial priority map therefore implies that the distance to the fovea plays only a minor role for guiding free-viewing gaze on average. Rather, the spatial priority of image locations in this dataset can be expressed as a scalar value that does not depend directly on the current location of the viewer's fovea—that is, DeepGaze III's spatial priority map is spatiotopic. Note that this conclusion may not hold for the first one or two fixations of a scanpath, before the viewer has a scene gist (Schütt et al., 2019), and also should not be taken to imply that a spatial priority map must be spatiotopically implemented in the brain.

In this article, we usually avoid the term “saliency” due to it is ambiguity. Especially in the computer vision literature, it commonly refers to all image-driven effects on gaze placement—whether described by simple or more complex features. This notion should be distinguished from the classic notion of saliency from psychology, which implies that features attracting attention consist of contrast and pop-out in simple image feature spaces such as luminance, color, or orientation. We have explored differences in fixation prediction between simple and more complex features previously (Kümmerer et al., 2017). In accordance with Zelinsky and Bisley (2015) and also other work by us (Kümmerer & Bethge, 2021), for our model, we here use the term “spatial priority” to denote all image-driven (and potentially task-modulated) effects on gaze placement.

Many scanpath models employ an explicit internal state representation that evolves over a scanpath and influences future fixation placement (e.g., Schütt et al., 2017; Sun et al., 2019; Yang et al., 2020). We specifically chose not give our model such an internal state representation. An internal state is a crucial component for a model that aims to implement a biologically plausible fixation selection mechanism. However, this is not what we try to achieve here. In this article, we are interested in predicting human scanpath behavior as well as possible and in understanding the relevance of different factors and interactions on fixation placement. An internal state would make many of the ablation studies that we conducted in this study impossible, for example, measuring how relevant earlier fixations are for gaze placement (Figure 7a).

### Outlook

Besides the analyses that we have presented here, we envision that DeepGaze III might enable interesting future research. For example, there might be effects present in the fixation selection process that are quite hard to find by analyzing scanpath data by hand. Deep learning might be able to pick up on such effects. While the model itself is not completely interpretable, it can be used to screen large datasets for interesting predictions where, for example, the predictions of DeepGaze III differ most from state-of-the-art mechanistic models such as SceneWalk. This can then inform new hypotheses about which effects might affect fixation selection. This approach for utilizing DeepGaze III is already the subject of ongoing work.

DeepGaze III so far uses information about only the previous four fixation locations of the scanpath history. Our analyses suggest that, at least for our datasets, this is enough to capture the most prominent effects of previous fixation locations. Nevertheless, at least for some tasks, most likely there are also effects from earlier fixation locations, and it would be worthwhile to extend DeepGaze III in a way that allows one to use these fixation locations. Recurrency might be a way to achieve this.

Besides fixation locations, fixation durations are also known to affect the selection of future fixation locations (Laubrock et al., 2013; Tatler et al., 2017; Nuthmann, 2017). So far, only few models such as SceneWalk and IOR-ROI-LSTM incorporate them, and we are planning on extending DeepGaze III to also make use of fixation duration information. This would allow us to quantify the relevance of fixation durations for fixation selection.

Although DeepGaze III performs better than all other models evaluated here, the model still has limitations. Figure 3 shows that DeepGaze III does not reproduce all statistical properties of scanpaths perfectly, and Figure 6b shows several failures to capture scanpath patterns in individual images. These limitations are most likely an effect of the readout architecture, which could not be made more powerful without creating overfitting issues on the present datasets. Larger datasets will make training even more powerful models feasible, allowing us an even more detailed picture of the different effects we analyzed here.

Finally, all analyses conducted here made use of free-viewing human scanpaths. Task and meaning are known to heavily influence viewing behavior (Yarbus, 1967; Land & Hayhoe, 2001; Rothkopf et al., 2007; Henderson, 2003; Öhlschläger & Võ, 2017), and we consider applying DeepGaze III in non-free-viewing situations one of the most interesting future research directions. In tasks like visual search, we expect there might be, for example, much more complicated interactions between scene content and scanpath history than the simple effects that we report here for free viewing. In the past, extending these models to other tasks was difficult due to the paucity of suitable large-scale datasets; we are hopeful for the future given recent datasets such as COCO-Search-18 (Chen et al., 2021; Yang et al., 2020). We are already planning to utilize these new datasets to extend DeepGaze and model the influence of task on human scanpaths.

We make the code and trained model parameters for DeepGaze III publicly available at https://github.com/matthias-k/DeepGaze.

### Conclusion

We present a new state-of-the-art model for human free-viewing scanpath prediction, DeepGaze III, which uses a structured deep learning architecture to achieve both high prediction performance and some degree of model interpretability. Using ablation studies, we show that on our main dataset fixation selection depends more on scene content than previous scanpath history. Beyond average prediction performance, DeepGaze III also reproduces noteworthy statistical properties of human scanpaths. It is sometimes claimed that deep learning–based models are limited in their capacity to create scientific insight (Marcus, 2018; Henderson et al., 2019). We argue that the results presented in this article provide an additional example (see also, e.g., Kietzmann et al., 2019; Cadena et al., 2019) of how deep learning can in fact be be used to yield scientific contributions that are not only relevant but that would also be difficult to accomplish without the recent advances in deep learning.

## Appendix

### Scanpath similarity scores on MIT1003

Here we report model scores using the scanpath similarity metrics ScanMatch (Cristino et al., 2010) and MultiMatch (Jarodzka et al., 2010), which are commonly used in many works on scanpath modeling. Because they can result in unreliable scores (models can systematically score higher than the ground truth data; see Kümmerer & Bethge, 2021), we do not include them in the main article and we point out that they should be used only with caution. As will become apparent in the following, the problem of the unreliable scores is indeed visible in our case. For ScanMatch, we use the MATLAB implementation by Cristino et al. (2010), available at https://seis.bristol.ac.uk/~psidg/ScanMatch/. For MultiMatch, we use the python reimplementation of the original MATLAB toolbox by Wagner et al. (2019), available at https://github.com/adswa/multimatch_gaze. For both ScanMatch and MultiMatch, we use the default parameters included in the respective implementations. Since neither we nor most other included models model fixation durations, we disable the duration component of MultiMatch by using identical durations for all fixations.

We compute scanpath similarity metrics in two different settings: In Table 5, for each human scanpath, one model scanpath is generated starting with the same initial fixation and length, and similarity between these two scanpaths is reported. As “interobserver consistency,” we report the mean similarity between two human scanpaths on the same image. There are two models that perform better than DeepGaze III: LeMeur16, which scores best in ScanMatch, and MASC, which scores best in MultiMatch. However, in the ScanMatch metrics, many models substantially outperform the interobserver consistency (e.g., LeMeur16 with 0.564 compared to interobserver consistency of 0.433), demonstrating that the problem outlined above is not just a theoretical possibility. In MultiMatch, the problem is less severe but still visible.

**Table 5.** Scanpath similarity scores on the MIT1003 dataset. We evaluate MultiMatch and ScanMatch scores on single sample performance: For each ground truth human scanpath, we compute similarity to a sampled scanpath starting with the same initial fixation and having the same number of fixations. Italic model names indicate baseline models; bold model names indicate the models presented in this article. Errors indicate standard deviation over eight repeated training runs. In all metrics, higher scores indicate better performance. Bold scores indicate best performance for that metric. Models are sorted by total MultiMatch score.

	MultiMatch					
Model	Total	Shape	Direction	Length	Position	ScanMatch
*Uniform*	0.765	0.847	0.650	0.802	0.762	0.350
STAR-FC	0.788	0.883	0.664	0.845	0.761	0.370
Itti&Koch (with WTA network)	0.794	0.865	0.679	0.828	0.805	0.411
SaltiNet	0.801	0.889	0.659	0.875	0.781	0.382
*Center bias*	0.825	0.902	0.671	0.892	0.834	0.454
Saccadic Flow	0.834	0.923	0.666	0.922	0.824	0.436
**DeepGaze III w/o Spatial Priority**	0.837 ± 0.000	0.925 ± 0.000	0.669 ± 0.001	0.921 ± 0.000	0.834 ± 0.001	0.451 ± 0.001
CLE	0.841	0.930	0.663	0.918	0.855	0.499
**DeepGaze III w/o Scanpath**	0.844 ± 0.001	0.913 ± 0.000	0.700 ± 0.001	0.902 ± 0.001	0.861 ± 0.001	0.520 ± 0.001
*Spatial Gold Standard*	0.845	0.911	0.704	0.899	0.865	0.527
SceneWalk	0.847	0.917	0.699	0.909	0.863	0.522
SceneWalk20	0.848	0.921	0.693	0.915	0.861	0.516
IOR-ROI-LSTM	0.856	0.918	0.743	0.908	0.855	0.501
**DeepGaze III**	0.858 ± 0.000	0.930 ± 0.000	0.710 ± 0.001	0.925 ± 0.000	0.868 ± 0.001	0.528 ± 0.001
LeMeur16	0.870	**0.938**	0.730	0.930	**0.884**	**0.564**
MASC	**0.873**	0.935	**0.743**	**0.933**	0.881	0.545
*Interobserver Consistency*	0.874	0.937	0.752	0.930	0.879	0.433

In Table 6, for each human scanpath, the most similar scanpath from all generated scanpaths on the same image is used for the score. This strategy is used in many studies. It can partially mitigate the problem that models can score systematically higher than the ground truth data, but it penalizes models less for producing very unrealistic scanpaths: As long as at least one reasonable scanpath per image is generated, the unrealistic scanpaths will not decrease the score. Here, as “interobserver consistency,” we report the mean similarity of a human scanpath to the most similar scanpath by another observer on the same image. In this setting, no model scores substantially higher than interobserver consistency (LeMeur16 reaches a ScanMatch score of 0.602 compared to 0.600 for interobserver consistency, but this is likely within the noise range). DeepGaze III is outperformed by the LeMeur16 model, which scores highest in all metrics.

**Table 6.** Scanpath similarity scores on the MIT1003 dataset. We evaluate MultiMatch and ScanMatch scores on best-match performance: For each ground truth human scanpath, we compute similarity to all sampled scanpaths on the same image (usually 15 scanpaths for 15 subjects; see section “Scanpath Statistics” for details) and report the score for the sampled scanpath that reaches highest similarity score. In the case of the MultiMatch score, we use the total score for selecting the best-matching scanpath. Italic model names indicate baseline models; bold model names indicate the models presented in this article. Errors indicate standard deviation over eight repeated training runs. In all metrics, higher scores indicate better performance. Bold scores indicate best performance for that metric. Models are sorted by total MultiMatch score.

	MultiMatch					
Model	Total	Shape	Direction	Length	Position	ScanMatch
STAR-FC	0.836	0.902	0.773	0.871	0.798	0.396
Itti&Koch (with WTA network)	0.841	0.890	0.769	0.866	0.839	0.418
*Uniform*	0.852	0.902	0.792	0.881	0.831	0.418
SaltiNet	0.875	0.924	0.813	0.914	0.848	0.457
*Center bias*	0.890	0.933	0.826	0.926	0.873	0.510
Saccadic Flow	0.897	0.944	0.830	0.939	0.873	0.515
IOR-ROI-LSTM	0.902	0.936	0.860	0.924	0.889	0.541
**DeepGaze III w/o Spatial Priority**	0.902 ± 0.000	0.947 ± 0.000	0.841 ± 0.001	0.941 ± 0.000	0.880 ± 0.000	0.527 ± 0.001
CLE	0.906	0.950	0.833	0.940	0.899	0.571
SceneWalk20	0.907	0.945	0.847	0.939	0.896	0.569
**DeepGaze III w/o Scanpath**	0.907 ± 0.000	0.943 ± 0.000	0.850 ± 0.001	0.935 ± 0.000	0.901 ± 0.000	0.574 ± 0.000
SceneWalk	0.908	0.944	0.849	0.937	0.900	0.573
MASC	0.908	0.946	0.846	0.940	0.901	0.562
*Spatial Gold Standard*	0.911	0.944	0.853	0.936	0.910	0.588
**DeepGaze III**	0.917 ± 0.000	0.952 ± 0.000	0.863 ± 0.001	0.946 ± 0.000	0.908 ± 0.001	0.590 ± 0.001
LeMeur16	**0.921**	**0.954**	**0.869**	**0.948**	**0.912**	**0.602**
*Interobserver Consistency*	0.932	0.958	0.888	0.953	0.927	0.600

### Performances per category on CAT2000

In Table 7, we show the performance of DeepGaze III as well as different ablated versions of the full model on each of the 20 categories in CAT2000. We quantify the effect of image content as the performance difference between the center bias model and the model without access to scanpath history. Furthermore, we quantify the effect of dynamcis as the performance difference between the center bias model and the model without access to image content.

**Table 7.** Prediction performance on the CAT2000 dataset by category as average log-likelihood in bit/fixation relative to a uniform model. We list model performance of the center bias model and ablated versions of DeepGaze III as well as the full DeepGaze III model. As “effect of image content,” we show the performance difference between the model without scanpath history and the center bias model. As “effect of dynamics,” we show the difference in performance between the model without image content and the center bias model. Categories are sorted by performance of the full DeepGaze III model.

Category	Center bias	W/o scanpath history	W/o image content	Full model	Effect of image content	Effect of dynamics
Jumbled	1.08	1.38	2.33	2.53	0.30	1.25
Satelite	1.18	1.31	2.56	2.64	0.14	1.38
Indoor	1.20	1.54	2.64	2.82	0.34	1.44
Cartoon	1.03	1.49	2.59	2.83	0.47	1.57
Inverted	1.32	1.61	2.70	2.85	0.28	1.38
OutdoorManMade	1.28	1.62	2.72	2.89	0.34	1.44
OutdoorNatural	1.44	1.69	2.78	2.92	0.26	1.35
Art	1.36	1.72	2.78	2.97	0.37	1.43
LineDrawing	1.41	1.64	2.87	2.98	0.23	1.45
Noisy	1.58	1.78	2.89	3.00	0.20	1.31
Fractal	1.60	1.79	2.92	3.04	0.20	1.33
Social	1.11	1.75	2.76	3.11	0.64	1.65
Pattern	1.75	1.88	3.10	3.17	0.13	1.35
Random	1.47	2.01	2.99	3.22	0.54	1.52
Object	1.56	2.02	3.05	3.26	0.46	1.49
Action	1.41	2.01	2.96	3.27	0.59	1.55
BlackWhite	1.64	2.08	3.10	3.30	0.44	1.45
Affective	1.44	2.06	3.01	3.32	0.62	1.57
LowResolution	1.93	2.06	3.29	3.34	0.12	1.36
Sketch	2.00	2.60	3.55	3.79	0.61	1.56
